# Supplementary material for: Positive Effects of Oral Antibiotic Administration in Murine Chronic Graft-Versus-Host Disease
Source: Int J Mol Sci. 2021 Apr 3;22(7):3745. doi: 10.3390/ijms22073745 (PMC8038334; doi:10.3390/ijms22073745)
Supplement: Supplementary file 1 [file ijms-22-03745-s001.pdf]

	cGVHD	+ GM	P value
Body weight loss	1.4 ± 0.49	0.2 ± 0.40	0.0053
Posture	1.2 ± 0.49	0.6 ± 0.49	0.094
Activity	1 ± 0.00	0.4 ± 0.49	0.040
Fur texture	1 ± 0.00	0.2 ± 0.40	0.0040
Skin integrity	0.6 ± 0.49	0 ± 0.00	0.040
Alopecia	0 ± 0.00	0 ± 0.00	
Diarrhea	1.6 ± 0.49	0 ± 0.00	0.00018

Table S1. Clinical signs of GVHD in non-treated and gentamicin-treated cGVHD mice (+GM): n = 5 per group. Data are presented as mean ± SEM. unpaired Student's t-test.

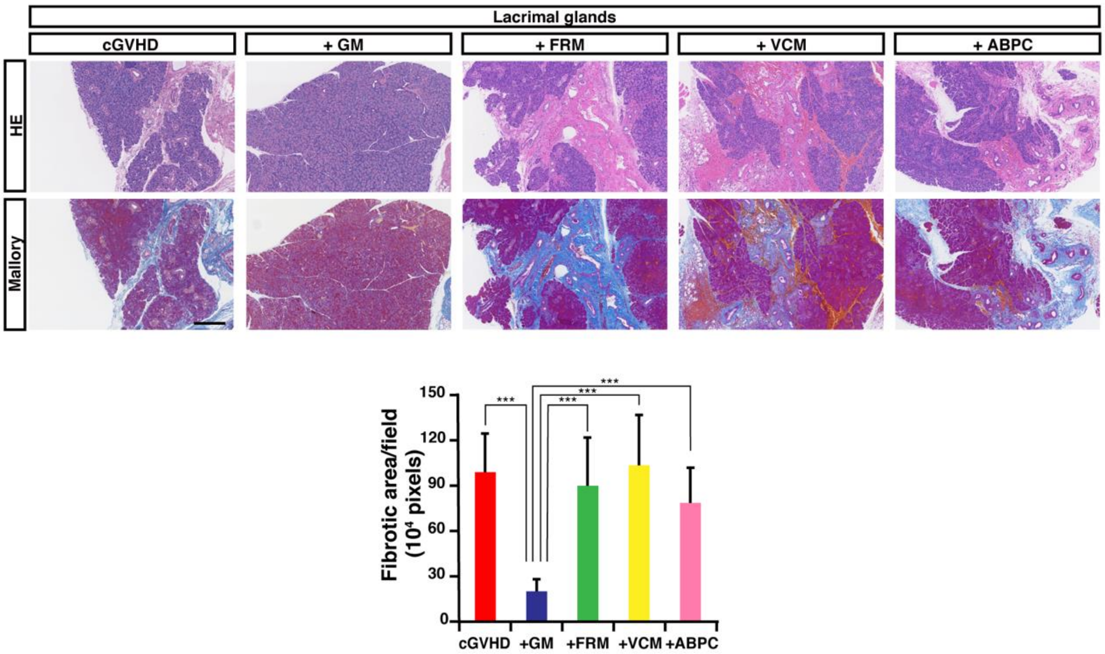

Figure S1. Pathological findings and analysis: H&E and Mallory staining of lacrimal glands (LGs) from non-treated cGVHD mice and gentamicin-treated cGVHD mice and antibiotics-treated cGVHD mice. GM, gentamicin; FRM, fradiomycin; VCM, vancomycin; ABPC, ampicillin. Blue fibrotic areas indicated by Mallory staining were measured using ImageJ (n = 5 per group; 3 fields). Data are presented as mean ± SEM. ***P <0.001, unpaired Student's t-test. Scale bar, 250 μm.

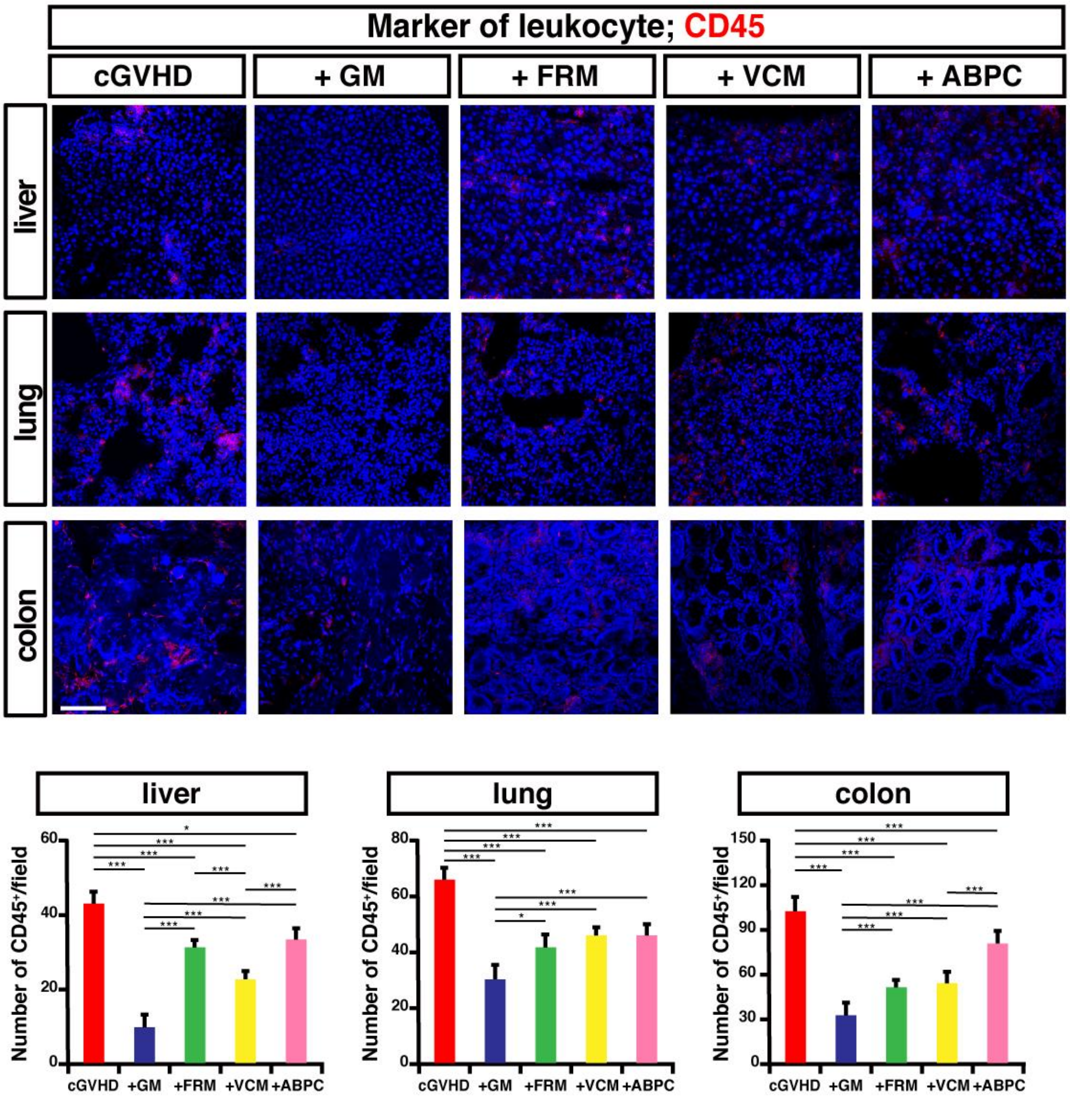

Figure S2. The expression of the leukocyte marker in cGVHD-targeted organs in antibiotics-tread cGVHD mice. Sections of cGVHD-targeted organs (livers, lungs, and colons) from non-treated cGVHD mice (n = 5, five fields per sample) and antibiotics-treated cGVHD mice (n = 5, five fields per sample) were stained for CD45 (leukocyte marker) (red). GM, gentamicin; FRM, fradiomycin; VCM, vancomycin; ABPC, ampicillin. Data are presented as mean ± SEM. **P* <0.05, ****P* <0.001, unpaired Student's *t*-test. Scale bar, 100 μm.
